# Ethnic Disparities and Inequalities in Dementia Care: An Extended Literature Review

**DOI:** 10.1192/bjo.2025.10090

**Published:** 2025-06-20

**Authors:** Anita Okeoghene Ighomereho

**Affiliations:** University of Nottingham, Nottingham, United Kingdom

## Abstract

**Aims:** Dementia is a leading cause of disability and loss of independence among older people. There is growing concern about ethnic disparities and inequalities in dementia care. In the UK, Black and South Asian people have a higher risk of developing dementia compared with their White counterparts. Despite this, there is underrepresentation of minority ethnic groups in dementia services and they are more likely to have a delayed diagnosis or no diagnosis.

This literature review aims to identify the ethnic disparities in dementia care. It also aims to explore the causes of delayed diagnosis and underdiagnosis of dementia amongst Black, Asian and Minority Ethnic (BAME) groups, particularly Black and South Asian ethnic groups in the UK.

**Methods:** An electronic literature search was performed using PubMed and Google Scholar.

**Results:** Across the literature, it is evident that health inequalities currently exist and exacerbate the disparities in the care of BAME dementia patients. These inequalities can affect quality of care and lead to overall poorer outcomes for people from BAME backgrounds.

Multiple risk factors for dementia such as hypertension and diabetes disproportionately affect Black and South Asian people.

Delayed diagnosis and underdiagnosis of dementia in BAME groups can be attributed to multiple barriers to accessing dementia diagnostic services such as stigma, lack of knowledge, misperceptions, language barriers and cultural beliefs.

In secondary care, patients from BAME groups are younger and have greater severity of dementia at the time of diagnosis.

Survival time following a dementia diagnosis can vary across different ethnic groups. However, there are contrasting findings between studies. Further research is required to investigate these contradictory findings to clarify how survival time post-diagnosis is affected by ethnic background.

Across multiple studies, data from large ethnic groups such as South Asian or Black are combined without taking into account the diverse subgroups within these larger groups. Further research is required within ethnic subgroups to provide a more person-centred approach.

**Conclusion:** There is a need to tackle the ethnic disparities in dementia care faced by South Asian and Black people in the UK. This requires action and collaboration between people from BAME communities, healthcare professionals and policymakers, in order to improve timely access to services. Further research should address the disparities to ensure equitable and inclusive dementia healthcare.

